# Eating vegetables before carbohydrates improves postprandial glucose excursions

**DOI:** 10.1111/dme.12073

**Published:** 2013-02-20

**Authors:** S Imai, M Fukui, N Ozasa, T Ozeki, M Kurokawa, T Komatsu, S Kajiyama

**Affiliations:** 1 School of Comprehensive Rehabilitation, Osaka Prefecture UniversityOsaka; 2 Department of Endocrinology and Metabolism, Kyoto Prefectural University of MedicineKyoto, Japan; 3 Department of Cardiovascular Medicine, Kyoto University, Graduate School of MedicineKyoto, Japan; 4 Doshisya Women's College of Liberal ArtsKyoto, Japan; 5 Kajiyama ClinicKyoto, Japan

Large fluctuations in blood glucose are reported to promote the micro- and macrovascular complications associated with Type 2 diabetes. Postprandial plasma glucose and glycaemic spikes are more strongly associated with atherosclerosis than fasting plasma glucose or HbA_1c_ level 1. Therefore, safe and effective interventions, including diet, are needed to reduce glycaemic variability and minimize hypoglycaemic events. The continuous glucose monitoring system is capable of detecting hypoglycaemia and hyperglycaemia that may be undetectable by self monitoring blood glucose and HbA_1c_ 2. In particular, the mean amplitude of glycaemic excursions is a significant determinant of overall metabolic control, as well as increased risk of diabetes complications.

We reported acute effects of eating vegetables before carbohydrates on postprandial glucose and insulin levels 3 and the long-term glycaemic improvements in patients with Type 2 diabetes 4. The method of the education included nutritional advice given in the form of a simple and easy meal plan of eating vegetables before carbohydrates. In order to reduce postprandial hyperglycaemia, patients were encouraged to consume every meal by eating vegetables prior to carbohydrates. In this study we evaluated whether eating vegetables before carbohydrates could reduce the daily postprandial glucose excursions assessed by continuous glucose monitoring system in Japanese patients with Type 2 diabetes and subjects with normal glucose tolerance.

Consecutive patients with Type 2 diabetes were recruited among outpatients regularly attending a diabetes clinic, the Kajiyama Clinic located in Kyoto, Japan, from 2011 to 2012. Diagnosis of diabetes was made according to the World Health Organization criteria. Confirmation of normal glucose tolerance was based on fasting blood glucose < 5.6 mmol/l and 2-h glucose concentration in an oral glucose tolerance test < 7.8 mmol/l. All participants were assigned to perform the continuous glucose monitoring system (CGMS, Medtronic Minimed Gold; Medtronic Minimed, Northridge, CA, USA) for 72 h by eating test meals of vegetables before carbohydrates and carbohydrates before vegetables on the 2nd and the 3rd day in a randomized crossover design. The test meals consisted of rice/bread, meat/fish and 500 g of vegetables, and contained 21 g of dietary fibre and 125.6 kJ kg^−1^ per day. The energy ratio of protein, fat and carbohydrates was 17, 25 and 58%, respectively. The subjects ate the first dish of vegetables for 5 min, then the main dishes, and consumed rice or bread with a 10-min interval between vegetables and carbohydrates in each meal, and then vice versa.

The glucose fluctuations were assessed by the following parameters obtained from the continuous glucose monitoring system and compared between the day of eating vegetables before carbohydrates and the day of eating the carbohydrates before the vegetables: the mean plasma glucose, standard deviation (sd), mean amplitude of glycaemic excursions and the largest amplitude of glycaemic excursions, postprandial plasma glucose, incremental area under the curve 0–3h (IAUC_0–3h_), and incremental glucose peak.

Nineteen outpatients with Type 2 diabetes [men/women 6/13; age 65.5 ± 9.4 years, duration of diabetes 16.4 ± 10.2 years; BMI 22.5 ± 3.1 kg/m^2^; HbA_1c_ 55.0 ± 10.9 mmol/mol (7.2 ± 1.0%); fasting plasma glucose 8.06 ± 2.67 mmol/l, diet/oral hypoglycaemic agents/insulin + oral hypoglycaemic agents 3/3/13; mean ± sd or *n*] and 21 subjects with normal glucose tolerance [men/women 2/19; age 29.8 ± 11.3 years; BMI 20.8 ± 3.0 kg/m^2^; HbA_1c_ 36.0 ± 6.6 mmol/mol (5.4 ± 0.6%); fasting plasma glucose 4.89 ± 0.50 mmol/l] were enrolled in the study. The levels of standard deviation, mean amplitude of glycaemic excursions, largest amplitude of glycaemic excursions, 1-h postprandial plasma glucose of breakfast, IAUC_0–3h_ of lunch and dinner, mean IAUC_0–3h_ and incremental glucose peak were significantly reduced when the participants ate vegetables before carbohydrates compared with the reverse regimen in both subjects with Type 2 diabetes and those with normal glucose tolerance; however, the values of mean plasma glucose were not different in either of the subject groups (Table 1). Two-hour postprandial plasma glucose levels of lunch and dinner, and IAUC_0–3h_ of breakfast were also significantly reduced in patients with Type 2 diabetes, while 1-h postprandial plasma glucose levels of lunch and dinner were significantly decreased in subjects with normal glucose tolerance. The reason for the reduction of postprandial plasma glucose levels by eating vegetables before carbohydrates can be explained, partly, by the dietary fibre content in the vegetables taken before the carbohydrates. Dietary carbohydrates consumed after vegetables were digested slowly and required less insulin for subsequent metabolic disposal 5. Other factors may influence the glycaemic response and digestion of carbohydrates in the small intestine, including the rate of digestion, cooking method, transit time and rate of intestinal absorption. Vegetables given before carbohydrates might stimulate incretin hormone secretion, which leads to the reduction in glycaemic excursions 6.

**Table 1:** Characteristics of glycaemic excursion in subjects with Type 2 diabetes and normal glucose tolerance

	Patients with Type 2 diabetes			Subjects with normal glucose tolerance		
		
	Vegetables before Carbohydrates	carbohydrates before vegetables	*P*	Vegetables before Carbohydrates	carbohydrates before vegetables	*P*
Mean plasma glucose (mmol/l)	8.01 ± 1.97	8.16 ± 1.90	NS	5.04 ± 0.37	5.22 ± 0.49	NS
Standard deviation (mmol/l)	1.69 ± 0.67	2.38 ± 1.13	< 0.01	0.69 ± 0.19	0.91 ± 0.38	< 0.01
Mean amplitude of glycaemic excursions (mmol/l)	4.36 ± 1.86	6.52 ± 3.17	< 0.01	1.56 ± 0.74	2.44 ± 1.09	< 0.01
Largest amplitude of glycaemic excursions (mmol/l)	6.82 ± 2.24	9.43 ± 3.98	< 0.01	3.10 ± 0.74	4.53 ± 1.66	< 0.01
1-h postprandial plasma glucose of breakfast (mmol/l)	9.53 ± 2.21	11.00 ± 3.54	< 0.05	5.92 ± 0.81	6.28 ± 1.06	< 0.05
2-h postprandial plasma glucose of breakfast (mmol/l)	9.66 ± 2.77	10.54 ± 3.27	NS	5.33 ± 0.54	5.21 ± 0.80	NS
1-h postprandial plasma glucose of lunch (mmol/l)	8.55 ± 2.78	9.78 ± 3.94	NS	5.62 ± 0.78	7.23 ± 1.58	< 0.001
2-h postprandial plasma glucose of lunch (mmol/l)	8.90 ± 2.96	10.84 ± 4.55	< 0.05	5.67 ± 0.95	5.95 ± 1.33	NS
1-h postprandial plasma glucose of dinner (mmol/l)	9.28 ± 2.44	10.17 ± 3.13	NS	5.68 ± 0.94	7.08 ± 1.59	< 0.01
2-h postprandial plasma glucose of dinner (mmol/l)	9.06 ± 2.50	10.48 ± 3.67	< 0.01	5.67 ± 0.78	6.19 ± 1.42	NS
IAUC_0–3h_ of breakfast (mmol/l)	321 ± 225	472 ± 331	< 0.05	114 ± 66	108 ± 54	NS
IAUC_0–3h_ of lunch (mmol/l)	397 ± 278	640 ± 421	< 0.05	155 ± 93	235 ± 135	< 0.05
IAUC_0–3h_ of dinner (mmol/l)	287 ± 260	532 ± 308	< 0.01	125 ± 93	229 ± 165	< 0.05
Mean IAUC_0–3h_ (mmol/l)	334 ± 254	546 ± 356	< 0.001	132 ± 85	191 ± 138	< 0.05
Incremental glucose peak of breakfast (mmol/l)	3.34 ± 1.63	5.08 ± 2.69	< 0.01	1.50 ± 0.63	1.97 ± 0.88	< 0.05
Incremental glucose peak of lunch (mmol/l)	3.10 ± 2.01	6.97 ± 3.53	< 0.05	1.67 ± 0.79	2.83 ± 1.40	< 0.01
Incremental glucose peak of dinner (mmol/l)	2.53 ± 1.84	4.71 ± 3.57	< 0.05	1.43 ± 0.76	2.66 ± 1.51	< 0.01
Mean incremental glucose peak (mmol/l)	2.99 ± 1.82	5.50 ± 3.34	< 0.001	1.56 ± 0.73	2.50 ± 1.33	< 0.001

In this study, we demonstrated that eating vegetables before carbohydrates reduced the postprandial glucose excursions compared with the reverse regimen in both subjects with Type 2 diabetes and those with normal glucose tolerance using continuous glucose monitoring system for the first time. The result of this study is important because eating vegetables before carbohydrates could be a novel method to reduce the incidence of cardiovascular disease 7–10. As one of the educational points in nutrition, the advice to patients with Type 2 diabetes should be to eat vegetables before carbohydrates, and this advice could even be applicable to healthy subjects in order to prevent future cardiovascular events.
